# Laying the Groundwork for Dementia Service Innovations: Developing and Validating Measures of Dementia Caregiving

**DOI:** 10.1093/geroni/igaf122.1567

**Published:** 2025-12-31

**Authors:** Yuanjin Zhou, Lauren Bangerter

## Abstract

Caregivers of persons living with dementia face significant challenges in managing their complex health and care needs, often due to insufficient training and support. A major barrier to addressing these challenges is the lack of targeted and validated measures that capture the complex process of dementia caregiving. This symposium presents five papers focused on developing and validating novel measures to enhance caregiver support and service innovation. The first paper introduces the development of the Caregiver Outcomes of Psychotherapy Evaluation, Individualized Needs and Goals (COPE-ING) assessment tool, which offers promise for rapidly identifying caregivers’ needs in clinical settings. The second paper aims to develop and establish the content validity of the Delirium Caregiving Self-Efficacy Scale using a Delphi Expert panel. This scale can be used to enhance dementia caregivers’ adherence to delirium prevention. The third paper describes the development and content validation of the Fall Risk Management Scale (FRMS), which can be used to engage dementia caregivers in fall risk management effectively. The fourth paper presents the data supporting the reliability and validity of a new Caregiving Style (CG-STYLE) measure. Findings support its utility in tailoring support to family caregivers’ unique caregiving styles. The fifth paper examines the structural validity of two clinical practical tools to evaluate person- and task-centered dementia mealtime care in nursing homes, with findings supporting the progression to establish these tools’ clinical utility. This discussant, Dr. Lauren Bangerter, will highlight the research and clinical implications of these novel measures, especially how they contribute to dementia service innovations.

